# Decreased self-reported receiving of social touch and social support predict loneliness in healthy adults

**DOI:** 10.1186/s41155-022-00228-w

**Published:** 2022-08-01

**Authors:** Cássia Regina Vieira Araújo, Bruna Eugênia Ferreira Mota, Rafaela Ramos Campagnoli, Vanessa Rocha-Rego, Eliane Volchan, Gabriela Guerra Leal Souza

**Affiliations:** 1 Laboratory of Psychophysiology, Department of Biological Sciences, Federal University of Ouro Preto, Ouro Preto, Brazil; 2 School of Nutrition, Federal University of Ouro Preto, Ouro Preto, Brazil; 3 Biomedical Institute, Fluminense Federal University, Niterói, Brazil; 4 Department of Neurobiology, Institute of Biology, Fluminense Federal University, Niterói, Brazil; 5 Institute of Biophysics Carlos Chagas Filho, Federal University of Rio de Janeiro, Rio de Janeiro, Brazil

**Keywords:** Loneliness, Social touch, Social support, Heart rate variability

## Abstract

Loneliness has emerged as a public health concern. Previous research has reported its deleterious effects on physical and mental health; however, some specific psychophysiological mechanisms in healthy adults remain to be elucidated. The aim of the current study is to investigate whether self-reported social support and social touch (giving and receiving social touch), as well as resting heart rate variability (HRV), are significant negative predictors of loneliness in healthy adults. The study sample consists of 120 healthy students (50% female) with a mean age of 21.85 years old (DP= 2.21). The students were asked to complete a psychiatric screening questionnaire utilizing loneliness, social support, and social touch scales. HRV was derived from an electrocardiographic signal recorded for 15 min, with the participant relaxed in a supine position. Linear regression analyses were conducted to evaluate loneliness as a function of social support, social touch (giving or receiving touch), and resting HRV. The results show that social support (*p*< 0.001) and social touch, specifically receiving touch (*p*< 0.002), accounted for a significant proportion of the variance in loneliness. However, neither giving touch nor resting HRV was a significant predictor of loneliness. The current study highlights specific psychosocial factors in healthy adults that should be considered as promising pathways in order to reduce or work toward preventing loneliness, thus promoting better health and well-being.

## Introduction

Many years of research have revealed the potential consequences of social isolation for human beings (see for review Holt-Lunstad et al., 2010; Holt-Lunstad et al., 2015). One of the first and most important findings was that social isolation constitutes a risk factor for mortality equivalent to or greater than obesity or cigarette smoking (House et al., 1988). As well as in the actual absence of social interactions, the perception of this absence has a significant impact on health. Thus, perceived social isolation is the concept of loneliness (Weiss, 1973). More complex than being isolated, loneliness means feeling alone, and it depends on the quality of the social network rather than the quantity of friends (Cacioppo & Patrick, 2008). Owing to the COVID-19 outbreak, in which social distancing and lockdowns were imposed, loneliness has become a more recent topic of interest. Nonetheless, even before the current pandemic, loneliness had already been reported as an emerging public health issue (Cacioppo & Cacioppo, 2018).

A broad body of research suggests that loneliness is a significant risk factor for the development of psychopathologies and other health impairments (Jung et al., 2019; Wang et al., 2020). Regarding mental health, an important association was found between loneliness and depression (Ge et al., 2017), chronic social stress (Hawkley et al., 2008), anxiety (Muyan et al., 2016), and psychosis (da Rocha et al., 2018). A causal relationship between loneliness and psychiatric disorders has also been suggested (Mushtaq et al., 2014).

It is not surprising that loneliness is an issue of increasing concern given that human beings are considered to be an ultra-social and hyperactive cooperative species (Tomasello, 2014). For several species, including primates, social touch represents a fundamental aspect in communication and plays an important role in maintaining social bonds and the cohesion of groups (Dunbar, 2010; Jakubiak & Feeney, 2017). Proximity and interpersonal social contact are prominent components for survival and well-being, from premature babies to the elderly (Charpak et al., 2017; Cruciani et al., 2021; Feldman & Eidelman, 2003). Indeed, recently, the C-tactile pleasant touch pathway, a specialized system underlying the processing of receiving social touch, was broadly described (Ackerley et al., 2014; Gazzola et al., 2012; Lloyd et al., 2013; Löken et al., 2009; Morrison et al., 2010). Much less attention has been given to the investigation of the benefits for giving touch; thus, we have made this inclusion within the present study. Maturana and Verden-Zöller (2008) proposed that human hands are caressing organs. The tactile exploration of pleasantness of surfaces’ involves vibration-sensitive Pacinian Corpuscles and proprioceptive afferents in hand palms (Klöcker et al., 2013). Gentsch et al. (2015) showed that touching others’ skin elicits sensory and haptic pleasure in the giver, possibly involving the same receptors as described by Klöcker et al. (2013). It has been shown that chronic loneliness is associated with a greater preferred interpersonal distance (Saporta et al., 2021) and that lonely individuals reported feeling social touch as less agreeable (Saporta et al., 2022, Cacioppo et al., 2010; Silva et al., 2017). Thus, delineating specific links between self-reported social touch (giving and receiving) and loneliness is a gap in the literature that remains to be investigated.

Subjective perceptions of being inserted into a support network may have relevant implications for loneliness. As such, studies have demonstrated that social support is an important variable in lowering loneliness (Bernardon et al., 2011; Deniz et al., 2005). For example, perceived friendship support was found to be the best predictor of lower loneliness scores (Pierce et al., 1991). Furthermore, perceived social support from family and friends was found to buffer against loneliness in the study by Schmitt and Kurdek (1985). Thus, in this study, we would like to corroborate the literature about social support and loneliness and add to the discussion on self-reported social support and its ability predict loneliness in healthy adult participants.

During the last few decades, psychophysiological research has used heart rate variability (HRV) to study social engagement (Porges, 2007; Shaffer & Ginsberg, 2017). HRV is a standard noninvasive tool for assessing the action of the autonomic nervous system over the heart based on variations in the RR interval between consecutive heartbeats (Shaffer & Venner, 2013; Smith et al., 2020). Importantly, HRV has been useful as a marker of pathological conditions (Beauchaine & Thayer, 2015). For example, it was demonstrated that a low HRV is associated with a higher risk of mortality (Kleiger et al., 2005), cardiovascular disease (Carnethon et al., 2002), obesity (Kageyama et al., 1997), depression (Kemp et al., 2010), anxiety (Servant et al., 2008), and chronic stress (Lampert et al., 2016). Given that loneliness is a risk factor for several diseases, HRV could be a prominent tool for investigation. However, few studies have investigated the relationship between HRV and loneliness, and the results have not converged. Some studies did not find an association between resting HRV and loneliness in young (Cacioppo et al., 2002; Muhtadie et al., 2015), middle-aged, or older adults (Hawkley et al., 2006; Muhtadie et al., 2015). On the other hand, other studies with larger samples showed a significant (Roddick & Chen, 2020) and modest (Hawkley et al., 2003) negative association between loneliness and resting HRV. Therefore, it is necessary to conduct more investigations using a healthy sample of both sexes to clarify these ambiguities.

Solving the question of loneliness is a major challenge, especially considering the occurrence of social isolation during the COVID-19 pandemic and the possibility of future pandemics. Cacioppo and Cacioppo (2018) argue that a solution to this challenge is possible if a collective effort is met. In the current literature, there remains a lack of data regarding the psychophysiological mechanisms underlying loneliness. Thus, the present study aims to investigate whether psychosocial factors, specifically self-reported social support and social touch (receiving and giving touch), and HRV while resting, a physiological indicator of trait of health and social functioning, could be predictors of loneliness in healthy adults.

## Methods

### Participants

The sample comprised of 120 undergraduate students (60 females) with a mean age of 21.85 years old (DP = 2.21). Participants were recruited according to the following criteria: age ranging from 18 to 30 years, being an undergraduate or graduate student, not being under medication (except for contraceptives), not having a diagnosis of psychiatric or heart disease, being a non-smoker, and not using alcohol or drugs with daily or almost daily frequency. The study protocol was approved by the Research Ethics Committee of the local institution and all participants provided written informed consent. Data were collected prior to the COVID-19 outbreak.

### Psychosocial assessment

Initially, participants completed a health and lifestyle questionnaire to evaluate data on age, sex, exercise practice, medication use, caffeine ingestion, and physical health. The mental health status of the participants was assessed using the Psychiatric Screening Questionnaire (PSQ) (Harding et al., 1980) translated and adapted to Portuguese (Mari & Williams, 1986). The PSQ consists of a scale composed of 20 items with “yes” and “no” options for responses, used to diagnose suspicion of common mental disorders. To analyze this questionnaire, all affirmative answers were added. Scores equal to or greater than five (for males) or seven (for females) indicate the presence of some mental disorder, and in this case, the participants were not included in the analysis.

Loneliness scores were assessed using the revised UCLA Loneliness Scale (Russell et al., 1980) translated and adapted to Portuguese (Neto, 1989). The Portuguese version of the revised UCLA Loneliness Scale is an 18-item questionnaire that evaluates the loneliness experience of an individual during different periods in time and their satisfaction with their social relations. This scale score ranges from 18 to 72 with items randomly alternated (nine items scoring from 1 = “never” to 4 = “several times” and nine items scoring from 1 = “several times” to 4 = “never”). The Cronbach’s alpha of the scale for this study was 0.89.

Social support was measured using the Social Support Scale (Chor et al., 2001) translated and adapted to Portuguese (Griep et al., 2005). The Social Support Scale is a 19-item questionnaire evaluating different aspects of social support (i.e., affective support, material support, emotional support, and positive social interaction). In this study, social support was used as the total score, which was the sum of all subscales. This scale’s score ranges from 19 to 95 (all items scoring from 1 = “never” to 5 = “always”), with a Cronbach’s alpha of 0.83.

Social touch was evaluated using the Mutual Grooming Scale (Nelson & Geher, 2007) translated to Portuguese (Campagnoli et al., 2015). This instrument consists of 12 items measuring the frequency of giving touch and 12 items measuring the frequency of receiving touch, both over the last 12 months. Participants rated each item on both subscales considering close individuals (i.e., family, intimate friends), therefore excluding strangers. Scores range from 14 to 98 (all items scoring from 1 = “never” to 7 = “one or more times/day”), both for the receiving and for the giving of touch. The Cronbach’s alpha for this scale was 0.81 for giving touch and 0.85 for receiving touch.

### Heart rate variability

Heart rate variability was accessed through electrocardiographic (ECG) signal processing. One PC-compatible computer was used for ECG acquisition using the Acknowledge (BIOPAC Systems Inc, Goleta, USA) software program. The signal recording was performed using reusable 8 mm electrodes (Ag/AgCl) in the 1st cardiac lead through an electrocardiograph ECG100C module coupled to the MP100 system (BIOPAC Systems Inc, Goleta, USA) at a sampling frequency of 1000 Hz with the participant relaxed in the supine position. We decided to measure HRV in the supine position because our record lasted 15 min, and we believed that it would be more comfortable and relaxing for the participant.

Data processing followed the recommendations of the Task Force of the European Society of Cardiology and the North American Society of Pacing Electrophysiology (Task Force of the European Society of Cardiology and the North American Society of Pacing Electrophysiology, 1996). We employed Kardia, a MATLAB (MathWorks Inc., MA) software toolbox, to analyze the cardiac parameters (Perakakis et al., 2010).

An offline peak detection algorithm (derivative plus threshold) was used to estimate the R-wave fiducial points, after which the series was screened manually and corrected for artifacts. Standard deviation of normal to normal of all intervals (SDNN) and root mean square of successive RR interval differences (RMSSD) were extracted using a time-domain analysis, whereas high-frequency (HF) and low-frequency (LF) components were extracted using a frequency-domain analysis, as recommended (Laborde et al., 2017; Task Force of the European Society of Cardiology and the North American Society of Pacing Electrophysiology, 1996). These indices reflect different aspects of autonomic nervous system functioning; RMSSD and HF are measures of parasympathetic activity (Ernst, 2017; Pentillä et al., 2001; Shaffer & Ginsberg, 2017). SDNN represents a global estimate of HRV with both sympathetic and parasympathetic influences (Shaffer & Ginsberg, 2017; Task Force of the European Society of Cardiology and the North American Society of Pacing Electrophysiology, 1996), and LF can be influenced by vagal, sympathetic, and baroreflex mechanisms (Del Paso et al., 2013; Goldstein et al., 2011; Task Force of the European Society of Cardiology and the North American Society of Pacing Electrophysiology, 1996).

### Procedure

Upon arriving at the laboratory, the participant was asked to fill out a questionnaire on health and general habits, the Psychiatric Screening Questionnaire (Mari & Williams, 1986), the revised UCLA Loneliness Scale (Neto, 1989), the Social Support Scale (Griep et al., 2005), and the Mutual Grooming scale (Campagnoli et al., 2015). Soon after, the electrodes to obtain the electrocardiogram (ECG) were placed on the 1st cardiac lead. The participant was instructed to rest in a supine position to register the ECG. The total time for ECG recording was 15 min, where the initial 5 min comprised of adapting the participant to the electrodes and the last 10 min consisted of the baseline test session itself**.** The room temperature was controlled by reverse cycle air conditioning equipment, which was turned on whenever the days were very hot or cold. In addition, the room temperature was monitored using a digital thermometer. The total duration of each experiment was approximately 1 h. All experiments were carried out from 8 am to 4 pm to avoid major HRV fluctuations (Sammito & Böckelmann, 2016).

### Statistical analyses

Statistical analyses were made using Statistica version 7.0 (StatSoft Inc., OK). Normality of data was evaluated using the Kolmogorov-Smirnov test. The HRV components were logarithm-transformed to fit data on a parametric distribution. In order to test the associations between loneliness and social variables, linear regression analyses were conducted in separate models. In each model, loneliness was entered as the dependent variable and other measures as predictors (social support in model 1, receiving touch in model 2, and giving touch in model 3). For HRV analyses, 17 individuals were excluded because of technical problems in the recording or signal processing. Thus, the final HRV analyses were performed using the data from 103 participants (53 females and 50 males) where loneliness was entered as the dependent variable and other measures as predictors (log SDNN in model 4, log RMSSD in model 5, log HF in model 6, and log LF in model 7), all controlled by age and sex (1−β= 0.99, *α*=0.05). The alpha level for statistical significance was set at 0.05. All tests were corrected for multiple comparisons using Bonferroni adjusted alpha levels of 0.007 per test.

## Results

Descriptive statistics of the sample are reported in Table 1.

**Table 1:** Descriptive statistics for psychosocial measures and HRV

Psychological measures	*n*	Minimum	Maximum	Mean	SD
Gender (female-male)	60-60				
Age (years)	120	18.0	28.0	21.9	2.2
PSQ score	120	0.0	7.0	3.3	2.1
Loneliness score	120	18.0	58.0	30.9	7.3
Social support score	120	42.1	100.0	81.8	13.0
Receiving touch score	120	14.0	75.0	35.2	14.5
Giving touch score	120	14.0	90.0	38.2	15.8
HRV	n	Minimum	Maximum	Median	P25–75
SDNN (ms)	103	16.1	111.8	55.4	40.7–71.0
RMSSD (ms)	103	8.1	130.7	43.9	30.6–67.4
HF (ms^2^)	103	6.3	1416.1	216.9	96.8–509.5
LF (ms^2^)	103	28.3	1196.1	290.4	125.2–470.0

*PSQ* Psychiatric Screening Questionnaire, *HRV* heart rate variability, *SDNN* standard deviation of the NN interval, *RMSSD* root mean square of successive differences, *HF* high-frequency component, *LF* low-frequency component

**Fig. 1:**
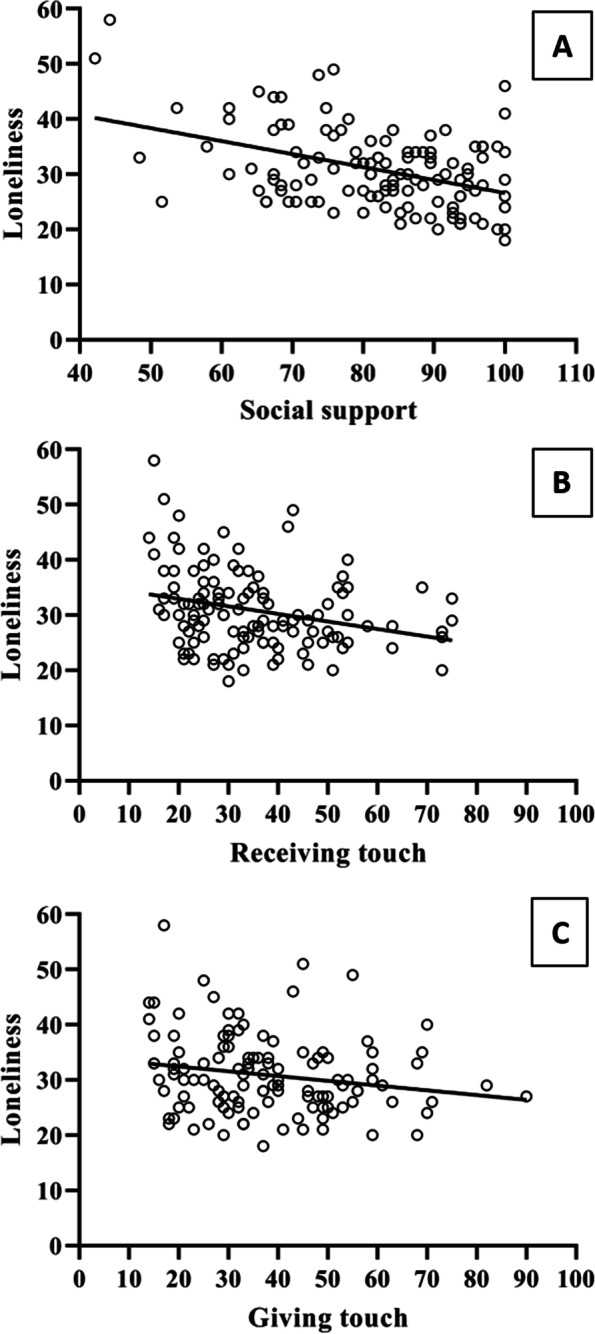
Graphic representation of regression analyses of loneliness and psychosocial assessments. **A** Regression analyses predicting loneliness as a function of social support (*Β*=− 0.42, *p* < 0.001). **B** Regression analyses predicting loneliness as a function of receiving touch (*Β*=− 0.27, *p* = 0.002). **C** Regression analyses predicting loneliness as a function of giving touch (*Β*= − 0.18, *p* = 0.04)

Linear regression analyses were performed to investigate whether psychosocial measures were significant predictors of loneliness. The results showed that social support explained 17% of the variance in loneliness (*R*^2^= 0.17, *F*(1,118)= 25.42, *Β*= −0.42, SE *B*= 0.08, *t*= −5.04, *p*< 0.001) (model 1) (Fig. 1A). Regarding self-reported mutual grooming, it was found that receiving touch accounted for 6.7% of the variance in loneliness (*R*^2^= 0.07, *F*(1,118)= 9.55, *Β*= − 0.27, SE *B*= 0.08, *t*= − 3.09, *p*= 0.002) (model 2) (Fig. 1B), whereas giving touch did not reach statistical significance (*R*^2^= 0.03, *F*(1,118)= 4.31, *Β*= −0.18, SE *B*= 0.09, *t*= −2.07, *p*=0.04) (model 3) (Fig. 1C).

**Fig. 2:**
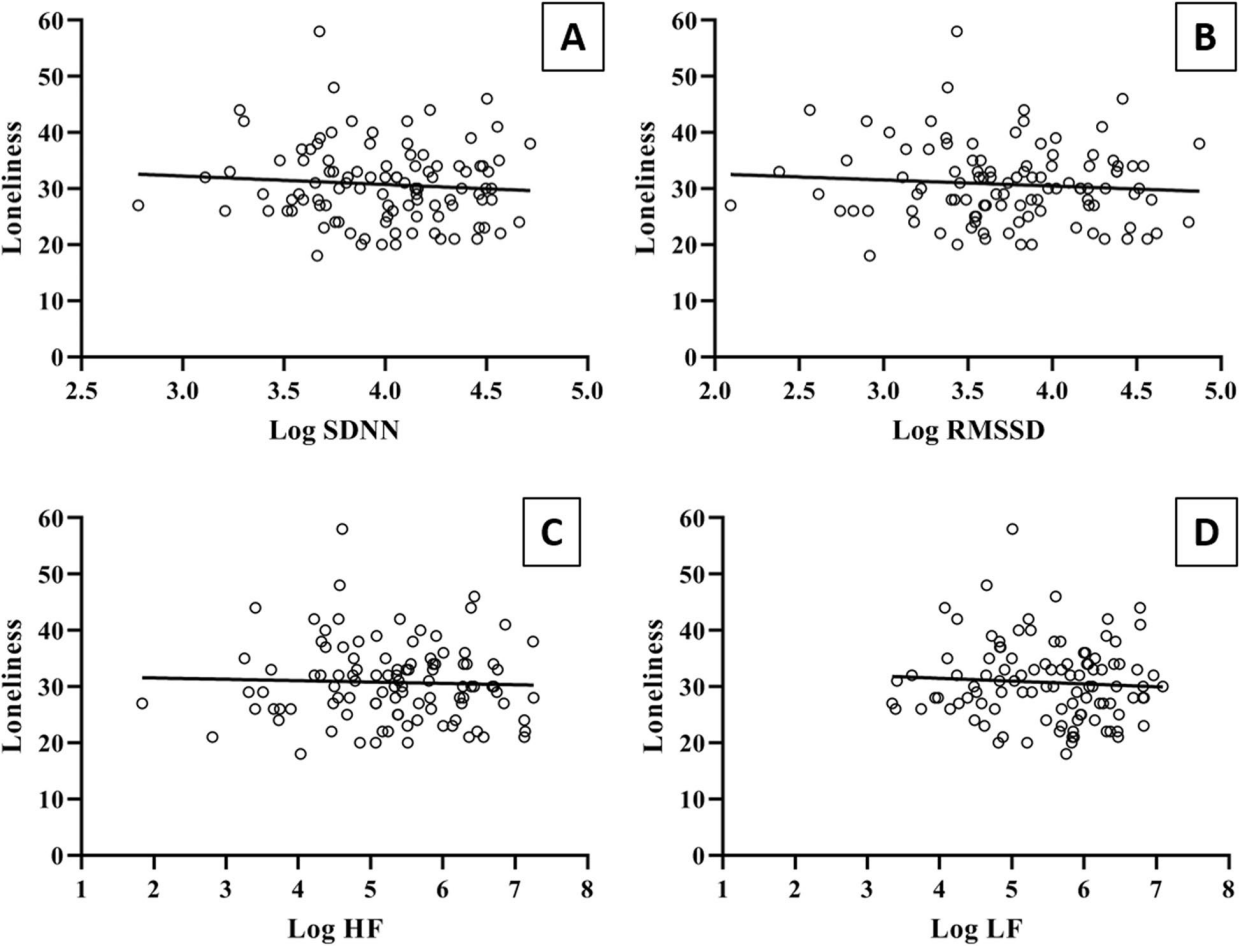
Graphic representation of regression analyses of loneliness and heart rate variability variables. **A** Regression analyses predicting loneliness as a function of SDNN (*Β*=− 0.12, *p* = 0.57). **B** Regression analyses predicting loneliness as a function of RMSSD (*Β*=− 0.11, *p* = 0.63). **C** Regression analyses predicting loneliness as a function of log HF (*Β*=− 0.05, *p* = 0.82). **D** Regression analyses predicting loneliness as a function of log LF (*Β*=− 0.10, *p* = 0.59)

Multiple linear regression analyses were conducted to test HRV components as predictors of loneliness. The results revealed that none of the components accounted for a significant variance in loneliness (see details in Table 2 and Fig. 2).

**Table 2:** Regression analysis predicting loneliness as a function of HRV

Model	Predictors	B	SE B	t	p
4	Age	− 0.02	0.10	− 0.17	0.86
	Sex	0.12	0.10	1.12	0.26
	log SDNN	− 0.12	0.11	− 1.15	0.25
5	Age	− 0.02	0.10	− 0.18	0.86
	Sex	0.10	0.10	0.99	0.32
	log RMSSD	− 0.11	0.10	− 1.04	0.30
6	Age	− 0.01	0.10	− 0.12	0.90
	Sex	0.09	0.10	0.86	0.39
	log HF	− 0.05	0.10	− 0.50	0.62
7	Age	− 0.02	0.10	− 0.18	0.86
	Sex	0.13	0.11	1.21	0.23
	log LF	− 0.10	0.11	− 0.99	0.32

Model 4: *R*^2^= 0.01, *F*(3,99)= 0.65, *p*= 0.57; model 5: *R*^2^= 0.01, *F*(3,99)= 0.57, *p*= 0.63; model 6: *R*^2^= 0.009, *F*(3,99)= 0.29, *p*= 0.82; model 7: *R*^2^= 0.01, *F*(3,99)= 0.63, *p*= 0.59

*SDNN* standard deviation of the NN interval, *RMSSD* root mean square of successive differences, *HF* high-frequency component, *LF* low-frequency component

## Discussion

The current study aimed to investigate whether self-reported social support, social touch, and components of HRV while resting are significant predictors of loneliness in a healthy sample of undergraduate students. Our results showed that loneliness significantly accounted for social support and social touch, specifically receiving touch. In contrast, neither giving touch nor resting HRV was a significant predictor of loneliness.

First, in support of previous literature findings, a significant association was found between loneliness and social support. This finding was also reported in a previous study by Lee and Goldstein (2016), which showed the effect of social support from friends on reducing levels of loneliness in a sample of undergraduate students. Early adulthood is characterized by several transitions (Goosbya et al., 2013). For example, many young people leave their families looking for opportunities to improve their careers, which was the case for the population investigated in the current study. This may be one reason as to why early adulthood is considered a period of greater vulnerability in experiencing loneliness. Such a fact raises the necessity of providing support and social inclusion to these vulnerable individuals. Additionally, lonelier people may feel less pleasure in social contexts (Silva et al., 2017) and have increased attention to negative social stimuli (Cacioppo et al., 2010). Thus, it could be argued that if the social environment seems less rewarding for lonely people, it may be an obstacle in searching for social interactions, reducing opportunities for creating and maintaining bonds. This may impact the possibility of receiving social support, which contributes to further increasing the feelings of loneliness.

In the present study, we provide new evidence in that self-reported receiving of decreased social touch during the last year (chronic effect) is a predictor of greater loneliness. Several studies have documented the beneficial effects of social touch in humans in experimental designs different from ours. For example, Coan et al. (2006) showed a reduced activation in some brain regions associated with emotional and behavioral threat responses when women held their husband's hand in comparison with not holding a hand. Ditzen et al. (2007) found that individuals who had physical contact during a stress task showed lower increases in cortisol levels and heart rate in comparison to the ones who had received verbal support or to the ones who had no support. There is also evidence that receiving a gentle touch reduces feelings of social exclusion (von Mohr et al., 2017). Heatley Tejada et al. (2020) showed that even in low-contact, individualistic societies receiving touch plays an important role in decreasing loneliness scores. Our findings corroborate with the literature and add the important insight of self-reported receiving of touch in the last year (chronic effect), which is different from receiving touch in a laboratory experimental session (acute effect) as already had been showed. This finding could be beneficial when considering how to buffer the deleterious effects of loneliness.

From an ethological perspective, the conscious perception of receiving touch may have interesting outcomes. For example, in some primate species, where allo-grooming plays a crucial role in communication, engagement in social activities depends on the amount of touch received (Dunbar, 2010). In humans, there is evidence from an experimental study evaluating close partners’ interactions that although both providing and receiving gentle touch were perceived as pleasant, being touched was more pleasant and significantly decreased heart rate, producing a calming effect (Triscoli et al., 2017). This could explain the significant result for receiving touch and the non-significant result for giving touch in the present study. Furthermore, there is a link between CT-fiber activation, which mediates gentle touch input, and oxytocin release during social interactions (Walker et al., 2017). Oxytocin also promotes an increase in vagal activity (Uvnäs-Moberg & Petersson, 2022), which is related to social functioning (Porges, 2007). Therefore, the link between the oxytocin system, vagal activity, and CT fibers may be another potential mechanism explaining the lack of social connection felt by lonely people. This may be a plausible mechanism underlying the decrease in loneliness upon receiving touch.

The present results indicate that touch is marginally significant as a predictor of loneliness. Of importance for the current discussion, a previous study showed that visual social stimuli promoted an accelerated reaction time in the flexion of the fingers, a motor task that bears resemblance with the social touch (Souza et al., 2012). Additionally, it was found that exposure to bonding pictures (with social touch cues) increased subjective feelings of sociability and activity of smile muscles (Mota et al., 2021) as well as the activity of muscles involved in a caress-like movement (Campagnoli et al., 2015). Furthermore, the authors provided evidence of decreased feelings of isolation after priming with bonding pictures and a reduction in the motor readiness potential amplitude preceding caress on a soft cloth (Campagnoli et al., 2015). Taken together, these findings are in line with the results of the present study, reinforcing the importance of social touch in promoting social bonding and thus decreasing loneliness, which is essential for human health and survival.

In this study, we did not find any evidence of resting HRV as a predictor of loneliness. Several studies have shown that a low HRV at rest is associated with a wide range of disorders and risk factors for several diseases (Beauchaine & Thayer, 2015; Carnethon et al., 2002; Kageyama et al., 1997; Kemp et al., 2010; Kleiger et al., 2005). In addition, a vast literature has outlined that some of these same pathological conditions are associated with loneliness (Cacioppo & Cacioppo, 2014; Mushtaq et al., 2014). As such, it would be expected an association between lower loneliness and higher resting HRV. In this study, we do not confirm this association. Roddick and Chen (2020), using a large healthy woman sample, showed a strong negative association between resting HRV and loneliness. The findings from Hawkley et al. (2003) supported a modest negative association. On the other hand, other studies did not find this association (Cacioppo et al., 2002; Hawkley et al., 2006; Muhtadie et al., 2015), which is similar to our findings. Possible reasons for why we did not find a significant negative association between resting HRV and loneliness is that our sample size may have been underpowered to detect the effect previously reported in the literature. Our study also differs from previous research as we included participants from both sexes, unlike Roddick and Chen (2020), and we recorded HRV in supine position, whereas Roddick and Chen (2020) and Hawkley et al. (2003) collected the HRV in seated position. Another point is that we used the Portuguese version of the revised UCLA Loneliness Scale that has 18-item, different from the English version that has 20-item. But as the questionnaire was translated and adapted to Portuguese (Neto, 1989), we believe that it did not influence the results.

Loneliness is a relevant topic that has increased in interest over the last decade and to an even greater extent following the outbreak of the coronavirus pandemic. In fact, an emerging body of research has reported the impact of imposed social distancing and loneliness on well-being and overall health (Bao et al., 2021; Clair et al., 2021; Cooper et al., 2021; Szwarcwald et al., 2021). As such, it is possible that the COVID-19 pandemic might have worsened a scenario that was already underway. Thus, implications of the worldwide expansion of loneliness must be further explored taking into account the effects of the current pandemic as well as other factors previously known to affect this condition.

It is important to note that there were some limitations in this study. Firstly, we recorded the ECG in a supine position which could change the influence of sympathetic and parasympathetic control to the heart. Secondly, we recorded the ECG for a long period of time, in which some participants could fall asleep. Thirdly, we did not record respiration as a variable. In turn, these limitations may affect the interpretability of the findings.

## Conclusion

The current study provides evidence that decreased self-reported social support and receiving of touch are important predictors of loneliness. These results highlight the effects of specific psychosocial factors that should be considered a promising pathway for reducing, or even preventing, loneliness, thus promoting better health and well-being.

## Data Availability

Data and survey materials will be made available upon request.

## Abbreviations

ECG: Electrocardiographic
HF: High-frequency component
HRV: Heart rate variability
LF: Low-frequency component
PSQ: Psychiatric Screening Questionnaire
RMSSD: Root mean square of successive differences
SDNN: Standard deviation of the NN interval
UCLA: University of California, Los Angeles
